# Malnutrition After Malabsorptive Metabolic Bariatric Surgery: When Should the Surgery Be Reversed?

**DOI:** 10.7759/cureus.97830

**Published:** 2025-11-26

**Authors:** Oday Al-Asadi, Mostafa Mahran, Danny Nguyen, Mikari Scipioni, Thakshyanee Bhuvanakrishna

**Affiliations:** 1 General Surgery, Homerton University Hospital, London, GBR; 2 General and Upper Gastrointestinal (GI) Surgery, Ain Shams University, Cairo, EGY; 3 Upper Gastrointestinal (GI) Surgery, Homerton University Hospital, London, GBR; 4 General Surgery, Queen Mary University of London, London, GBR; 5 Bariatric Surgery, Homerton University Hospital, London, GBR

**Keywords:** bariatric surgery, excess weight loss, liver failure, malnutrition, metabolic bariatric surgery (mbs), reversal

## Abstract

Background: Metabolic bariatric surgery (MBS) is the most successful way to tackle obesity and its related diseases. However, long-term side effects can arise. One rare but serious complication is severe protein-energy malnutrition, which may need surgery to reverse the effects on gut absorption function.

Objectives: We aimed to analyze the clinical, anthropometric, and biochemical criteria, as well as the operative outcomes for patients who underwent reversal surgery, and to assess the preoperative parameters that led to the decision to reverse bariatric surgery in patients with malnutrition.

Methods: A retrospective cohort study was conducted on all patients who underwent MBS over a five-year period (from July 2018 to July 2023) at the bariatric tertiary unit of Homerton University Hospital in London, UK.

Results: From a total of 645 cases that underwent MBS, nine (1.4%) patients required some form of reversal procedure due to severe malnutrition. All patients exhibited severe hypoalbuminemia (mean albumin level of 17.2 g/L), iron deficiency anemia (mean hemoglobin level of 104.67 g/L), and micronutrient deficiencies. They also presented with excess weight loss (mean percent excess weight loss (%EWL) of 119.3%). This necessitated admission for parenteral nutrition and trace elements infusion to optimize the patients' condition before reversal/revision surgery. Eight out of nine patients showed improvement after reversal/revision surgery, while one patient remained malnourished. The decision to offer reversal surgery and the type of surgery performed were based on local individual case assessments and multidisciplinary team (MDT) discussions.

Conclusions: Severe malnutrition following bariatric surgery is rare (1.4%) but serious. Reversal procedures are effective treatment options; however, they should be performed by an expert team in tertiary centers. While certain clinical features were commonly observed in our patient series, these findings cannot be generalized due to study limitations. Nonetheless, they can help inform future research. The question of when to perform reversal procedures remains unresolved, underscoring the need for larger, multicenter studies to better define indications and optimal timing.

## Introduction

Bariatric surgery is the most effective method for weight loss; however, it can be associated with significant long-term side effects. One of these rare but recognized complications is protein-energy malnutrition [1]. These deficiencies may require the reversal of the bariatric surgery, which has been shown to be effective in improving nutritional status. Such procedures should be performed not only by skilled surgeons but also by structured multidisciplinary bariatric teams in quality hospitals equipped with an ICU, interventional radiology, and surgical endoscopy teams [2].

The reported complication rate for reversal is higher than for primary procedures, reaching up to 29% [1]. These complications may include early issues such as anastomotic leaks, sepsis, and bleeding, as well as late complications, most notably weight regain and de novo gastroesophageal reflux [1]. Due to its rarity, there are no established guidelines for determining whether a patient requires reversal surgery [2].

The present study aimed to analyze the clinical, anthropometric, and biochemical criteria, as well as operative outcomes for those who underwent reversal operations, and to assess the preoperative parameters that led to the decision for reversal of bariatric surgery in patients with malnutrition.

## Materials and methods

A retrospective cohort study was conducted on all patients who underwent metabolic bariatric surgery (MBS) over a five-year period at the bariatric tertiary unit of Homerton University Hospital in London, UK.

All cases that underwent malabsorptive bariatric surgery from July 2018 to July 2023 were reviewed, and those reversed due to malnutrition (nine patients) were included. Patients who were reversed for other reasons, such as chronic abdominal pain, non-healing marginal ulcers, neuroglycopenia, or severe dumping syndrome, were excluded from this study. Patients who underwent restrictive procedures, such as sleeve gastrectomy (SG) or adjustable gastric band, were also excluded.

Other causes of malnutrition were ruled out through relevant investigations and consultations with a gastroenterologist as needed. Cases of bacterial overgrowth or pancreatic exocrine insufficiency were treated with the appropriate antibiotics or pancreatic exocrine extracts, respectively.

These nine cases were further analyzed in detail, including clinical, anthropometric, and biochemical parameters, type of primary procedure, degree of weight loss, details of hospital admissions, requirements for parenteral nutrition and trace element infusions, as well as the presence of organ failure and other life-threatening complications. Key laboratory values used to confirm the patients' malnutrition status included albumin, liver and kidney function tests, hemoglobin, iron, folate, copper, zinc, selenium, vitamin A, vitamin B12, vitamin D, calcium, and phosphate. The data were extracted from the hospital's electronic patient record (EPR) system.

All cases were discussed at the bariatric multidisciplinary team (MDT) meeting before surgery. The decision for reversal was made after optimizing the patient's nutritional state to minimize perioperative complications. A wider MDT comprising bariatric surgeons, bariatric physicians, clinical biochemists, gastroenterologists, nutritionists, dietitians, bariatric-specialized nurses, psychologists, anesthetists, and intensivists was involved in managing these very complex cases. The patients were involved in the decision-making process, and informed consent was obtained formally before surgery.

Data were analyzed using Microsoft Excel 2024 (Microsoft Corp., Redmond, USA). Continuous variables were presented as means or medians, while categorical variables were expressed as numbers (n) and percentages (%). Follow-up was conducted via telephone and face-to-face at outpatient clinics.

## Results

From a total of 645 cases that underwent MBS, nine (1.4%) patients required some form of reversal procedure due to severe malnutrition.

Patient demographics

The patients' demographics and comorbidities are listed in Table 1. The median age for the nine patients was 46 years, with seven (77.8%) identified as female. Of the patients, six (66.7%) were Caucasian and three (33.3%) were African American.

**Table 1:** Demographic and clinical characteristics of the patients MN = malnutrition

Variable	Patient 1	Patient 2	Patient 3	Patient 4	Patient 5	Patient 6	Patient 7	Patient 8	Patient 9	Total No.	%	Median
Age at reversal (years)	68	43	57	57	46	61	25	44	40	-	-	46
Female (n)	0	1	1	1	1	0	1	1	1	7	77.8	-
Time from diagnosis of MN to reversal (months)	9	24	3	3	2	1	6	7	4	-	-	4
Race												
Caucasian (n)	1	1	0	1	1	1	0	1	0	6	66.7	-
African American (n)	0	0	1	0	0	0	1	0	1	3	33.3	-
Asian (n)	0	0	0	0	0	0	0	0	0	0	0	-
Other (n)	0	0	0	0	0	0	0	0	0	0	0	-
Comorbidity												
Cardiovascular disease (n)	0	0	0	0	0	0	1	1	1	3	33.3	-
Pulmonary disease (n)	1	0	0	1	1	0	1	1	0	5	55.6	-
Kidney disease (n)	0	0	0	0	0	0	1	0	0	1	11.1	-
Liver disease (n)	0	0	0	1	1	0	0	0	0	2	22.2	-
Diabetes mellitus (n)	1	0	0	0	0	0	0	0	0	1	11.1	-
Psychiatric disease (n)	0	0	0	0	0	1	0	0	0	1	11.1	-

Clinical and biochemical measurements

The laboratory data are presented in Table 2. The serum albumin was lower than normal for all patients, with a mean albumin level of 17.2 g/L (range 9-29). The mean hemoglobin level was 104.67 g/L (range 92-116). Serum iron levels were also generally low, with a mean level of 6.55 µmol/L (range 4-9).

**Table 2:** Blood panel findings in the malnutrition stage --- = results not found; ALT = alanine aminotransferase; ALP = alkaline phosphatase; Hb = hemoglobin

Variable	Patient 1	Patient 2	Patient 3	Patient 4	Patient 5	Patient 6	Patient 7	Patient 8	Patient 9	Mean	Median
Serum albumin (g/L)	29	18	21	20	17	17	9	8	16	17.2	17
Serum bilirubin (µmol/L)	5	22	9	3	55	11	18	11	20	17.1	11
ALP (U/L)	174	151	102	139	148	82	150	120	116	131.3	139
ALT (U/L)	41	64	49	31	28	23	30	61	99	47.3	41
Hb (g/L)	99	111	92	112	100	111	103	98	116	104.7	103
Serum iron (µmol/L)	9	9	7	7	4	5	7	7	4	6.5	7
Folate	11.7	2.8	12	5.4	11.1	15.1	9.4	4.3	12.5	9.3	11.1
Urea (mmol/L)	4.7	2	5.1	5.1	2.1	7.2	6.6	10	5	5.3	5.1
Creatinine (µmol/L)	36	71	71	51	39	62	55	48	42	52.8	51
Copper (µmol/L)	10.8	22.4	9.6	15.3	3.3	8.9	7.3	2.9	14.7	10.6	9.6
Zinc (µmol/L)	7.8	7.5	6	8	4.5	7.5	8.2	2.3	8.9	6.7	7.5
Selenium (µg/L)	---	5.1	44	43	20.7	31.2	51.8	70	76.6	42.8	43.5
Vitamin A (µmol/L)	0.26	0.85	0.43	1.28	---	0.35	0.62	---	1.05	0.7	0.73
Vitamin B12 (pmol/L)	2000	2000	906	481	3306	2000	2000	1634	1624	1772.3	2000
Vitamin D (nmol/L)	39	49	95	73	115	15	91	87	64	69.7	73
Calcium (mmol/L)	2.17	2.22	1.97	2.1	2.05	1.99	1.99	1.66	2.15	2.0	2.1
Phosphate (mmol/L)	1.12	1.37	0.98	1.6	0.87	0.93	1.09	1.08	1.05	1.1	1.1

Regarding trace metals, the mean copper level was low at 10.6 µmol/L (range 2.9-22.4), and the mean zinc level was also low at 6.7 µmol/L (range 2.3-8.9). Similarly, despite one missing patient result, the selenium level was low, with a mean of 42.8 µg/L (range 5.1-76.6).

In two patients, results for vitamin A were not available; however, it was low in all the remaining seven patients, with a mean of 0.7 µmol/L (range 0.26-1.28). Hypocalcemia was observed in almost all patients, with a mean calcium level of 2 mmol/L (range 1.66-2.22), while vitamin D and phosphate levels were within normal ranges at 69.7 nmol/L and 1.1 mmol/L, respectively. The mean vitamin B12 level was high at 1772.3 pmol/L. 

Although deranged in some patients, the mean results for liver and kidney function tests were only mildly affected.

Table 3 shows the clinical presentation of patients in the malnutrition stage. All patients presented with significantly severe symptoms requiring prolonged hospital admission. Eight (88.9%) out of nine patients presented with bilateral leg edema. Diarrhea of five or more times per day, fatigue, and muscle wasting were observed in equal frequencies, with seven (77.8%) out of the nine patients affected.

**Table 3:** Clinical presentations at admission in the malnutrition stage Av(d) = average in days; DVT = deep vein thrombosis; N = not present; TPN = total parentral nutrition

Variable	Patient 1	Patient 2	Patient 3	Patient 4	Patient 5	Patient 6	Patient 7	Patient 8	Patient 9	Total No.	%	Av(d)
Leg edema (n)	0	1	1	1	1	1	1	1	1	8	88.9	-
Diarrhoea ≥ 5 (n)	1	1	0	0	1	1	1	1	1	7	77.8	-
Muscle wasting (n)	0	1	1	0	1	1	1	1	1	7	77.8	-
Fatigue (n)	1	1	1	0	1	1	0	1	1	7	77.8	-
Pre-reversal nutrition (TPN) (n)	1	1	1	1	1	1	1	1	1	9	100	-
Length of stay for pre-reversal nutrition (days)	21	49	58	51	53	31	30	58	57	408	-	45.3
Trace element infusion (n)	1	1	1	1	1	1	1	1	1	9	100	-
DVT (n)	N	N	N	N	N	1	N	1	1	3	33.3	-
Organ failure (n)	N	1	N	N	1	N	N	N	N	2	22.2	-

All nine patients (100%) required parenteral nutritional support and intravenous infusion of trace elements (including iron, folate, vitamins, and trace metals).

Three (33.3%) patients developed deep vein thrombosis (DVT), and two (22.2%) developed life-threatening complications due to organ failure, requiring admission to the ICU. The first case involved liver injury secondary to malnutrition, leading to hypotension, hypoglycemia, and acute kidney injury (AKI). This progressed to respiratory failure secondary to pneumonia and pulmonary edema, requiring intubation and high levels of vasopressor support. Subsequently, renal replacement therapy was initiated. The patient received appropriate antibiotics, starting with ceftriaxone and metronidazole, escalating to tazocin with gentamicin, and then to meropenem due to a highly resistant *Pseudomonas *infection.

The second patient experienced deranged liver function and confusion due to severe malnutrition, progressing to encephalopathy, AKI, and sepsis. This patient had a positive blood culture for *Propionibacterium*, which was treated with vancomycin, acyclovir, ceftriaxone, and amoxicillin.

The average length of stay for pre-reversal supportive treatment and optimization was 45.3 days.

Anthropometry

The average weight for the nine patients before the index malabsorptive bariatric operation was 139.5 kg, with an average body mass index (BMI) of 49.3 kg/m^2^. The lowest BMI reached was 21.6 kg/m^2^ on average. The mean percent excess weight loss (%EWL) was 119.3%. Following preoperative optimization and nutritional support, the average BMI before reversal surgery was 24.1 kg/m^2^. 

The follow-up BMI one year after the reversal surgery was 31 kg/m^2^ in seven out of the nine patients (two patients did not complete their follow-up) (Table 4).

**Table 4:** Anthropometric measurements DNA = did not attend the follow-up appointments; BMI = body mass index; %EWL = percent excess weight loss

Variable	Patient 1	Patient 2	Patient 3	Patient 4	Patient 5	Patient 6	Patient 7	Patient 8	Patient 9	Average
Weight before malabsorptive surgery (kg)	120	114	145	130	129	208	177	130	103	139.5
BMI before malabsorptive surgery (kg/m^2^)	45.7	39.7	47.8	53.4	42	67	71	44.9	32	49.3
Lowest BMI (kg/m^2^)	24.8	18	22	21	20.7	24	23	23.2	18	21.6
BMI before reversal surgery (kg/m^2^)	22.9	23.5	23.95	26.3	22	24.5	29	23.5	22	24.1
%EWL pre-reversal	104.2	131.8	111.3	112.9	95	112	103.6	114.2	189.2	119.3
BMI one year after reversal surgery (kg/m^2^)	33	<1 year	29	34	DNA	31	34	33.4	23	31

Surgical procedure and reversal

Table 5 presents the various primary procedure types as well as the types of reversal or revision procedures. Apart from one operation that required open conversion, all procedures were performed laparoscopically.

**Table 5:** Operative history details SASI = single anastomosis sleeve ileal bypass; RYGB = roux-en-Y gastric bypass; OAGB = one anastomosis gastric bypass; SG = sleeve gastrectomy; DS = duodenal switch; LL = limb lengthening; CC = common channel length

Patient	Index Operation	Revision (Partial Reversal) Operation	Reversal Operation	Access
					Patient 1	SASI	-	SG	Laparoscopic
Patient 2	RYGB	Feeding gastrostomy	Normal anatomy	Laparoscopic
Patient 3	OAGB	-	Normal anatomy	Laparoscopic
Patient 4	OAGB	-	Normal anatomy	Laparoscopic
Patient 5	DS	LL (CC from 1-2.5 m)	-	Laparoscopic
Patient 6	DS	LL (CC from 1-3 m)	-	Laparoscopic
Patient 7	DS	LL	SG	Open
Patient 8	DS	LL	SG	Laparoscopic
Patient 9	DS	LL (CC from 1-1.5 m)	-	Laparoscopic

Following the reversal operation, no major morbidity or mortality was observed. One patient required readmission within the first month after surgery. The average duration of hospital admission after surgery was 9.3 days.

Six out of the nine patients completed the two-year follow-up period. One patient completed only the one-year follow-up, while the remaining two patients did not attend any further follow-up appointments beyond the first few months after surgery. Among those who had a two-year follow-up, one patient developed dysphagia and esophageal dysmotility after the reversal surgery. Another patient required emergency surgery for duodenal perforation following endoscopic dilatation of a duodeno-ileal stricture. One patient remained malnourished (Table 6).

**Table 6:** Outcomes after reversal surgery DNA = did not attend minimum of one-year follow-up appointments; N/A = not applicable

Variable	Patient 1	Patient 2	Patient 3	Patient 4	Patient 5	Patient 6	Patient 7	Patient 8	Patient 9	Average
Major postoperative morbidity (at 30 days)	0	0	0	0	0	0	0	0	0	-
Postoperative mortality (at 30 days)	0	0	0	0	0	0	0	0	0	-
Readmission (at 30 days)	0	0	0	0	0	1	0	0	0	-
Length of stay after reversal (days)	3	3	9	8	17	8	10	14	12	9.3
Follow-up (years)	>2	DNA	>1	>2	DNA	>2	>2	>2	>2	-
Long-term complications/outcome	No	N/A	No	No	N/A	Yes	Yes	No	Yes	-

## Discussion

The most significant findings were hypoalbuminemia and iron deficiency anemia, which were seen in all nine patients. Albumin levels were very low (mean 17.2 g/L), indicating severe malnutrition. The findings of protein malnutrition and hypoalbuminemia are consistent with the available literature [3-5]. Despite the fact that they all received full oral dietary support, these patients failed to meet their nutritional requirements. Therefore, 9/9 (100%) patients required admission to the hospital for supervised parenteral nutrition. In addition, all nine (100%) patients required intravenous infusions of vitamins and trace elements, as they developed severe trace element deficiencies of varying combinations (including iron, folate, vitamin A, zinc, copper, and selenium) depending on the type of deficiency each had.

These patients typically required extended periods of admission. The average duration of admission for completion of these supportive treatments before reversal surgery was 45.3 days (range 21-58).

Liver and kidney functions were more affected in some patients compared to others. Their derangement can sometimes affect other blood test results, posing added difficulties in interpretation. Hypocalcemia might indicate poor bone health.

Vitamin B12 levels remained good for all patients, as they received three monthly injections regularly after surgery, making them unaffected by diminished gastrointestinal absorption.

Eight out of nine (88.9%) patients presented with bilateral leg edema, which varied in severity, being either confined to the legs or extending to involve the whole body. Diarrhea of five or more times per day was frequently observed in seven out of nine (77.8%) patients. This can be a very unpleasant symptom that considerably impacts the quality of life of patients. Fatigue and muscle wasting were also seen in similar frequencies (77.8% each).

Five patients developed potentially life-threatening events. Two (22.2%) experienced liver failure and sepsis, necessitating admission to the ICU, while three (33.3%) developed DVT involving major or proximal veins, requiring an extended period of treatment with enoxaparin.

Except for one patient, the preoperative BMI figures indicated that the patients were appropriately selected for bariatric surgery, with a mean BMI of 49.3 kg/m^2 ^(range 32-71). The lowest pre-reversal BMI mean was 21.6 kg/m^2^, with only two out of nine patients falling into the underweight category (below 18.5 kg/m^2^). Although this may be partly ameliorated by the presence of ascites and edema, it suggests that patients can become malnourished even if they are not classified as underweight. The mean %EWL was 119.3%, which is higher than expected figures following these operations [6,7].

All cases were approved for surgery by our bariatric MDT. The indications for reversal were either severe malnutrition refractory to nutritional and medical treatments and/or the presence of life-threatening events.

Operative details and outcomes

One patient with roux-en-Y gastric bypass (RYGB) and two with one-anastomosis gastric bypass (OAGB) were completely reversed to normal anatomy, as the gastric remnant was still preserved. Those who had single anastomosis sleeve ileal bypass (SASI) or duodenal switch (DS) were reversed to SG.

Three out of nine DS patients underwent limb lengthening (LL) only. Two of them, who had their common channel (CC) extended from 1 m to 2.5 m and 3 m, managed to resolve their malnutrition. The remaining patient had the CC extended to only 1.5 m and is still malnourished, requiring frequent hospital admissions for supportive treatment. This is likely due to the short CC, and the patient is not keen on undergoing further LL or reversal surgery.

Despite the complex clinical scenario for these cases, all patients recovered well after surgery, achieving a high success rate. There were no major morbidity or mortality reports in the first 30 days after surgery. Eight out of nine patients had complete resolution of malnutrition. This success is attributed not only to surgical expertise but also to the holistic approach of the wider MDT.

The average length of stay after surgery was 9.3 days (range 3-17). One patient was readmitted within the first 30 days, but no major intervention was needed.

A minimum follow-up period of two years was achieved in six out of nine patients, with 1/9 completing only one follow-up year and 2/9 not attending their follow-up appointments after completing the early recovery period in the first few months.

When should MBS be reversed?

There is no uniform definition in the bariatric literature for protein-energy malnutrition, its severity grading, or the threshold for revisional surgery [8]. The indications vary across different studies. Eilenberg et al. offered reversal surgery due to excess weight loss of 110.6% and liver dysfunction [9]. Sánchez-Pernaute et al. reversed 17 patients out of 333 who underwent the SADI procedure due to severe hypoalbuminemia and diarrhea of five times per day [3].

Abdelsalam et al. presented their single-center series of 10 patients with RYGB and OAGB, noting that the main indications for bypass reversal were malnutrition (33%), excess weight loss (29%), followed by chronic abdominal pain, chronic anemia, diarrhea, non-healing marginal ulcers, and persistent reflux, each representing 10%. Malnutrition was reflected by a low albumin level of 29.5 [4].

Aeschbacher et al. indicated in their study that the cause for reversal was often multifactorial, but the most common indications were malnutrition (53%), intractable diarrhea (42%), and hypoglycemia/dumping syndrome (37%) [10]. These studies and other similar research address malnutrition as one of the causes for the reversal of bariatric surgery; however, they did not assess the detailed criteria for patients with malnutrition.

Current clinical practice guidelines from major bariatric societies, including the British Obesity and Metabolic Surgery Society (BOMSS), International Federation for the Surgery of Obesity (IFSO), American Society for Metabolic and Bariatric Surgery (ASMBS), and European Society for Clinical Nutrition and Metabolism (ESPEN), do not provide clear, detailed instructions for bariatric surgery reversal due to malnutrition. ESPEN explicitly notes the lack of acceptable guidelines on the treatment of protein malnutrition after bariatric surgery [11-15].

In our series, we noticed that certain criteria were repeatedly observed in all or most of our patients, as shown in Table 7. These criteria were noted in the majority of our patients regardless of the type of primary malabsorptive procedure - whether it was SASI, RYGB, OAGB, or DS. While these criteria cannot be generalized due to the small number of cases in a single-center study, they can guide future larger collaborative studies to develop better-validated diagnostic tools, such as scoring systems.

**Table 7:** Common and serious clinical characteristics in patients with malnutrition after bariatric surgery PN = parenteral nutrition; %EWL = percent excess weight loss; BMI = body mass index; DVT = deep vein thrombosis

Category	Variable	Frequency Seen in Our Study, n (%)	Notes
Symptoms	Fatigue	7 (77.8%)	-
	Diarrhoea	7 (77.8%)	Watery or steatorrhea
Signs	Muscle wasting	7 (77.8%)	-
	Bilateral leg edema	8 (88.9%)	-
Anthropometry	Low BMI (<25)	9 (100%)	Difficult to put a cut-off figure, but in our series, all were below 25 kg/m^2^
	High %EWL (>100%)	8 (88.9%)	Difficult to put a cut-off figure, but in our series, the majority were above 100%
Deficiency	Hypoalbuminemia	9 (100%)	Severe
	Anemia	9 (100%)	Mostly iron-deficiency
Severity	Requirement of PN	9 (100%)	In-patient, supervised
	Requirement of trace elements infusion	9 (100%)	In-patient, supervised
Major event	DVT	3 (33.3%)	This could also include pneumonia or pathological fractures
Life-threatening event	Organ failure/sepsis	2 (22.2%)	For example: liver, heart, lungs, and kidneys

Study limitations

The study has some limitations that must be acknowledged. Firstly, it included a small series of cases, and the results cannot be generalized. Second, it is a retrospective cohort study that lacks randomization. Furthermore, the variability in the type of primary procedure (SASI, RYGB, OAGB, or DS) and the type of reversal (to normal, to gastric sleeve, or LL only) makes interpretation difficult and is subject to selection bias. Lastly, the laboratory tests were not fully comprehensive, with some missing data that could affect the results.

## Conclusions

Severe malnutrition following bariatric surgery is a rare (1.4%) but serious and potentially life-threatening complication. Reversal procedures are effective treatment options; however, they require a highly experienced MDT approach. Certain clinical features were commonly observed in our patient series, but these findings cannot be generalized due to the limitations of our study. Nonetheless, they can help guide further research collaboration. The question of when to perform a reversal remains unresolved, highlighting the need for larger, multicenter studies to better define the indications and optimal timing.

## References

[1] Dutta N, Scott AW, Marka NA, Wise ES, Amateau SK (2023). Endoscopic reversal of roux-en-Y gastric bypass prevents worsening of nutritional outcomes in patients with severe malnutrition. Front Gastroenterol.

[2] Verboonen Sotelo JS, Romero Manzano J, Vega Tostado G, Guzmán Barba JA, Esparza Estrada I, Orozco Álvarez Malo JO, González Ojeda A (2023). One-anastomosis gastric bypass reversal due to severe malnutrition and acute hepatic failure: a case report. J Surg Case Rep.

[3] Sánchez-Pernaute A, Lasses B, Antoñanzas LL (2024). Revisional surgery for malnutrition after SADI-S: prevalence, indications, techniques and outcomes. Updates Surg.

[4] Abdelsalam A, Ghobashy A, Elhawary R, Shenouda M, Khaled A, Abdellatif A (2025). Laparoscopic reversal of gastric bypass: a retrospective review. Obes Surg.

[5] Martins Tde C, Duarte TC, Mosca ER, Pinheiro Cde F, Marçola MA, De-Souza DA (2015). Severe protein malnutrition in a morbidly obese patient after bariatric surgery. Nutrition.

[6] Palumbo P, Banchelli F, Miloro C (2023). Weight loss trend after bariatric surgery in a population of obese patients. Clin Nutr ESPEN.

[7] MacVicar E, Lucocq J, Geropoulos G, Lamb PJ, Robertson AG (2025). The role of preoperative weight loss interventions on long-term bariatric surgery outcomes: a systematic review. J Clin Med.

[8] Abu-Abeid A, Goren O, Eldar SM, Vitiello A, Berardi G, Lahat G, Dayan D (2022). Revisional surgery of one anastomosis gastric bypass for severe protein-energy malnutrition. Nutrients.

[9] Eilenberg M, Langer FB, Beer A, Trauner M, Prager G, Staufer K (2018). Significant liver-related morbidity after bariatric surgery and its reversal - a case series. Obes Surg.

[10] Aeschbacher P, Garcia A, Frieder J (2025). Outcomes of reversal of malabsorptive and maldigestive bariatric procedures: a single center experience and a systematic review. Surg Obes Relat Dis.

[11] (2014). BOMSS guidelines on peri-operative and postoperative biochemical monitoring and micronutrient replacement for patients undergoing bariatric surgery. BOMSS.

[12] (2016). Appendix 8 guidance for clinical commissioning groups (CCGs): clinical guidance: revision surgery for complex obesity. https://www.england.nhs.uk/wp-content/uploads/2016/05/appndx-8-revision-surgery-ccg-guid.pdf.

[13] Eisenberg D, Shikora SA, Aarts E (2023). 2022 American Society of Metabolic and Bariatric Surgery (ASMBS) and International Federation for the Surgery of Obesity and Metabolic Disorders (IFSO) indications for metabolic and bariatric surgery. Obes Surg.

[14] Salminen P, Kow L, Aminian A (2024). IFSO consensus on definitions and clinical practice guidelines for obesity management - an international Delphi study. Obes Surg.

[15] Bischoff SC, Ockenga J, Eshraghian A (2023). Practical guideline on obesity care in patients with gastrointestinal and liver diseases - joint ESPEN/UEG guideline. Clin Nutr.

